# PINK1 import regulation; a fine system to convey mitochondrial stress to the cytosol

**DOI:** 10.1186/s12915-017-0470-7

**Published:** 2018-01-10

**Authors:** Shiori Sekine, Richard J. Youle

**Affiliations:** Biochemistry Section, Surgical Neurology Branch, National Institute of Neurological Disorders and Stroke, National Institutes of Health, Bethesda, Maryland 20892 USA

## Abstract

Insights from inherited forms of parkinsonism suggest that insufficient mitophagy may be one etiology of the disease. PINK1/Parkin-dependent mitophagy, which helps maintain a healthy mitochondrial network, is initiated by activation of the PINK1 kinase specifically on damaged mitochondria. Recent investigation of this process reveals that import of PINK1 into mitochondria is regulated and yields a stress-sensing mechanism. In this review, we focus on the mechanisms of mitochondrial stress-dependent PINK1 activation that is exerted by regulated import of PINK1 into different mitochondrial compartments and how this offers strategies to pharmacologically activate the PINK1/Parkin pathway.

## Overview of PINK1/Parkin-dependent mitophagy

**Fig. 1.**
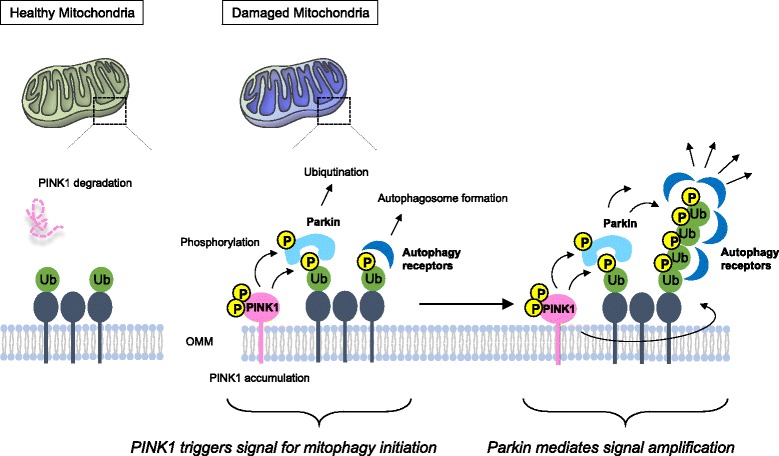
An overview of PINK1/Parkin-mediated mitophagy. In damaged mitochondria that lose mitochondrial membrane potential (ΔΨm), PINK1 import is blocked, which results in the accumulation of full-length PINK1 on the outer mitochondrial membrane (*OMM*). The TOM complex ensures the correct positioning of dimeric PINK1, and PINK1 kinase activity becomes activated through auto-phosphorylation. PINK1 phosphorylates ubiquitin, which triggers recruitment of Parkin recruitment to mitochondria and activation of its E3 ligase activity. At the same time, phospho-ubiquitin recruits autophagy receptors to initiate autophagosome formation. Parkin acts as an enhancer of this signaling through further ubiquitination of mitochondrial proteins

PTEN-induced putative kinase 1 (PINK1) is a mitochondrial Ser/Thr kinase that was identified as an autosomal recessive gene for familial recessive early-onset Parkinson disease (PD) in 2004 [1]. In 2006, important genetic studies in *Drosophila melanogaster* stemming from the original Parkin mutant fly discovery [2] suggested that PINK1 shares a common pathway with the E3 ubiquitin ligase Parkin, another autosomal recessive gene product of PD, where Parkin apparently functions downstream of PINK1 [3, 4]. PINK1 is targeted to mitochondria [1], whereas Parkin is located in the cytosol [5, 6]. The difference in subcellular localization of each protein posed the question of how and where those two proteins worked together. In 2008, it was identified that Parkin is recruited to mitochondria upon mitochondrial damage induced by genetic or chemical depolarization of mitochondria, and mediates the autophagic elimination of damaged mitochondria [7]. Subsequently, it was found that Parkin recruitment to damaged mitochondria requires PINK1 that accumulated on the outer mitochondrial membrane (OMM) in response to mitochondrial damage [8–11]. Thus, PINK1 and Parkin cooperate to maintain a healthy mitochondrial network by promoting autophagic elimination of damaged mitochondria (Fig. 1). In early papers, mitochondria were often stressed with uncoupling agents such as CCCP. More recent reports show that mitochondrial-specific OXPHOS inhibitors (e.g., oligomycin and antimycin A), forced mitochondrial ROS generation using novel techniques (e.g., mtKillerRed [12]), and even misfolded proteins in the mitochondrial matrix—as discussed later—also induce PINK1/Parkin-dependent mitophagy, emphasizing that various stresses can activate this pathway. In addition, recent papers show that PINK1 and Parkin are involved in other types of mitochondrial quality control such as mitochondrial-derived vesicles (MDVs) and even immune responses by mitochondrial antigen presentation [13].

Following identification of PINK1/Parkin-dependent mitophagy, the identification of ubiquitin as a substrate of PINK1 [14–16] advanced the understanding of the events that occur downstream of PINK1 activation (Fig. 1). PINK1 phosphorylates ubiquitin attached to OMM-localized proteins. At least two E3 ligases, such as Mul1 and March5, reside on the OMM [17–19], and some OMM-localized proteins are constitutively ubiquitinated by these E3 ligases, in part to mediate protein turn-over. Therefore, PINK1 likely targets these pre-existing ubiquitin molecules on mitochondria. Ubiquitin phosphorylated by PINK1 recruits Parkin to damaged mitochondria, and the E3 ligase activity of Parkin is activated by binding to phospho-ubiquitin [20, 21]. PINK1 also phosphorylates Parkin’s ubiquitin like domain, which stabilizes it in an active state [22, 23]. Substrate specificity of Parkin seems to be relatively low, but the above mechanisms can restrict Parkin activation to damaged mitochondria. In addition, phosphorylated ubiquitin can trigger autophagosome formation through the recruitment of autophagy receptors [24, 25], although cell free systems do not reveal selective binding of autophagy receptors to phospho-ubiquitin [24–26]. Activated Parkin ubiquitinates several mitochondrial substrates on the OMM, leading to the enrichment of ubiquitin molecules around damaged mitochondria [27]. These poly-ubiquitin chains are phosphorylated by activated PINK1, which creates a positive feedback amplification cycle on damaged mitochondria. Thus, PINK1 accumulated on the mitochondrial surface communicates with cytosolic molecules through phosphorylating ubiquitin in order to signal mitochondrial damage to the cytosol. How does PINK1 accumulate on the OMM in response to mitochondrial damage? In the following, we will summarize the mechanisms of PINK1 activation as a mitochondrial stress sensor. Downstream events of PINK1 activation, including Parkin activation and subsequent autophagosome formation, have been recently reviewed [28, 29].

## PINK1 import in healthy mitochondria

### Basic hypothesis of PINK1 import machineries

The majority of mitochondrial proteins, including PINK1, are encoded in nuclear DNA. These proteins are translated in the cytosol as precursors and transported into mitochondria. Mitochondria are double-membrane organelles, and imported proteins are delivered to multiple mitochondrial sub-compartments. This complicated sorting is achieved by several import machineries depending on the specific amino acid sequences displayed by the precursor proteins [30].

**Fig. 2.**
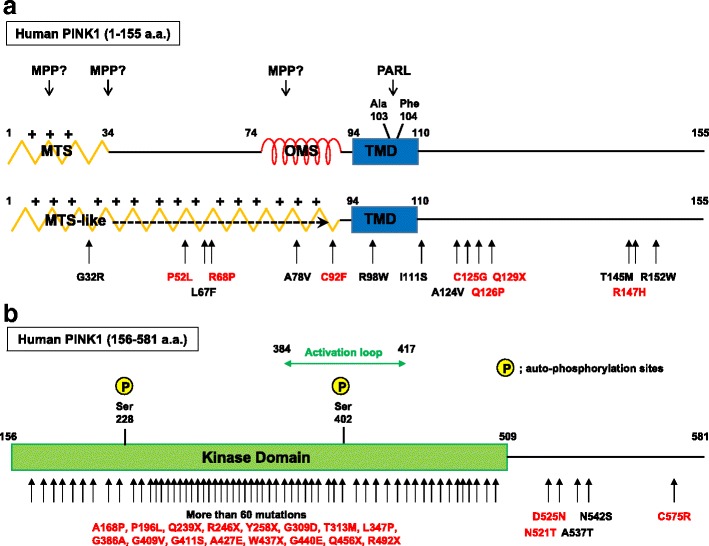
The domain structure of human PINK1. **a** The N-terminus of PINK1 (amino acids 1–155) contains three important domains for the determination of sub-mitochondrial localization; the mitochondria targeting sequence (*MTS*), the transmembrane domain (*TMD*), and the newly identified outer mitochondrial membrane localization signal (*OMS*) (*upper panel*). However, MTS could be longer (*lower panel*), and the precise cleavage sites of MPP have not been determined. PARL cleaves between Ala103 and Phe104 within TMD. **b** PINK1 has a kinase domain in its C-terminus (amino acids 156–581). Ser228 and Ser402 are auto-phosphorylation sites of PINK1. *Arrows* indicate PD-associated mutations. Mutations in *red* have been experimentally verified as loss of function mutations [86]. Most of the mutations reside in the kinase domain

**Fig. 3.**
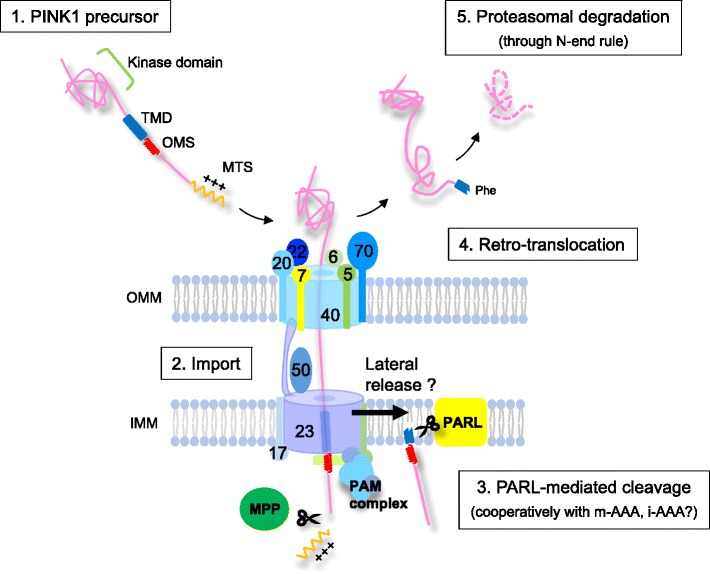
PARL-mediated cleavage of PINK1. After passing through the Tim23 complex, MPP cleaves the N-terminal MTS of PINK1 and PARL cleaves PINK1 within the TMD. It is not known how PARL can gain access to the PINK1 TMD during import. Cleaved PINK1 is retro-translocated into the cytosol and constitutively degraded by the proteasome via the N-end rule pathway. PARL-mediated PINK1 cleavage may also be supported by other mitochondrial proteases such as m-AAA and i-AAA

It was originally reported that PINK1 has a mitochondrial targeting sequence (MTS) in its N-terminus (amino acids 1–34 amino acids) [1] (Fig. 2a, upper panel). MTS-carrying precursor proteins are imported into mitochondria through the OMM-localized TOM (translocase of the outer membrane) complex and the inner mitochondrial membrane (IMM)-localized Tim (translocase of the inner membrane) 23 complex [30] (Fig. 3). The TOM complex consists of surface receptors (Tom20, Tom22, Tom70), the translocation pore (Tom40), and small accessary subunits (Tom7, Tom6, Tom5). Following recognition of the MTS by Tom20 cooperating with Tom22, precursors are guided into the Tom40 channel and transferred to the Tim23 complex in the IMM. Translocation of the positively charged MTS through the Tim23 complex is energetically driven by the electrical membrane potential (ΔΨm) across the IMM. After passing through the Tim23 translocase, the N-terminal MTS domain reaches the matrix, where the MTS is cleaved off by the matrix-localized protease, MPPα/β. This pathway is called the “presequence pathway”, and is considered to be involved in the targeting of most matrix-localized proteins and in some IMM- and intra-membrane space (IMS)-localized proteins. After passing through the Tim23 complex, matrix-localized proteins are further pulled into the matrix by the ATP-consuming import motor complex, the so-called PAM complex. Some types of hydrophobic segments following the MTS act as “stop-transfer” signals, which promote the arrest of some precursors during the import process and their lateral transfer into the lipid bilayer of the IMM. Soluble IMS proteins are often generated through additional cleavage from the laterally inserted precursors. Thus, stop-transfer through the Tim23 complex is one of the pathways responsible for sorting of both IMM- and IMS-localized proteins.

The MTS of PINK1 was originally annotated to amino acids 1–34 [1], and when a fluorescent protein (e.g., GFP) was fused with the first 34 amino acids of PINK1, several groups showed that PINK1 (1–34)-GFP localized in mitochondria [31, 32]. More detailed analysis confirmed that PINK1 (1–34)-GFP is imported into the matrix [33]. Domain prediction analysis revealed that PINK1 has a hydrophobic transmembrane (TM) domain (amino acids 94–110) in addition to the MTS (1–34) [34] (Fig. 2a). When the sequence around this TM domain is deleted, the PINK1 mutant appears to be localized in the matrix, confirming that the TM domain of PINK1 acts as a stop-transfer as predicted [33, 35].

Collectively, from these structural and experimental observations, PINK1 import under steady state conditions appears to follow the conventional stop-transfer pathway.

### Mysterious aspects of PINK1 MTS

As discussed above, it is convincing that the N-terminal 34 amino acids of PINK1 themselves have an ability to act as a typical MTS. However, it is reported that the N-terminal region of PINK1 has several potential MPP cleavage sites [36], and the precise MPP cleavage site of PINK1 has not been determined (Fig. 2a, lower panel).

Moreover, mitochondrial targeting of PINK1 has more mysterious aspects. The typical MTS has the potential to form an amphipathic helix with one hydrophobic and one positively charged face [30]. Although the MTS has no consensus in primary sequence, several prediction programs allow one to identify putative MTS based on the above structural features [30]. Through these structure prediction analyses, several reports point out that the PINK1 MTS-like sequence can be stretched over a relatively long region, possibly from 1 to 98 amino acids of PINK1, which includes almost the whole N-terminal region up to the TM domain [36] (Fig. 2a, lower panel). Consistent with this, in a previous study, PINK1Δ34 still maintained the ability to localize to mitochondria [35]. In contrast, if the whole N-terminal region of PINK1 before the N-terminus of the TM domain is deleted, PINK1Δ91 loses mitochondrial localization, raising the possibility that mitochondrial targeting of PINK1 does not solely rely on the N-terminal 34 amino acids [35]. A recent paper from Matsuda’s group provided one answer to these observations [33], as will be described later in detail (see Section 4).

## PINK1 cleavage and degradation in healthy mitochondria

### PARL-mediated PINK1 cleavage

Several early reports noted that over-expressed PINK1 yielded a lower molecular weight (MW) band around 52 kDa in addition to the 64 kDa full-length form [31, 32, 34, 35, 37]. Because the 52 kDa band disappeared when Tim23 complex-mediated import is blocked by adding mitochondrial uncoupler (as mentioned above, Tim23 complex requires ΔΨm to import MTS-carrying precursors) [34, 37], it had been expected that the 52 kDa form was produced by an unknown mitochondrial protease in an import-associated manner. From a genetic study using *Drosophila melanogaster*, the mitochondrial protease called Rhomboid-7 was identified as a possible candidate mediating PINK1 activation by cleavage [38]. Subsequently, we and several other groups reported that its mammalian homologue, PINK1/PGAM5-associated rhomboid-like protease (PARL; although PARL originally was named Presenilin-associated rhomboid-like, a recent proposal more accurately renamed it based on its substrates [39]), can cleave PINK1 [40–42] (Fig. 3) to produce the 52 kDa form of PINK1. PARL knockout (KO) mouse embryonic fibroblasts (MEFs) display a 60 kDa form of PINK1 (slightly lower than full-length PINK1) [40], indicating that MTS-cleaved PINK1 accumulates in PARL KO MEFs under steady state conditions.

PARL is an IMM-resident protease that belongs to the rhomboid protease family [43]. Rhomboid proteases are intramembrane proteases that catalyze cleavage within or adjacent to TM domains within lipid bilayers [44]. To allow efficient cleavage within the lipid bilayer by rhomboids, helix-destabilizing residues such as Pro and Gly have been suggested to facilitate local helix unfolding or kinking of substrate TM domains [45]. In addition, it has been reported that rhomboids recognize specific amino acid sequences surrounding the cleavage site of their substrates [46], including small amino acid residues, such as Ala, Gly, Cys, and Ser, in the P1 position (just before the cleavage site). Indeed, EDMAN degradation analysis revealed that PARL cleaves PINK1 between Ala103 and Phe104 [47]. These two amino acids seem to be evolutionarily conserved in PINK1 among vertebrates [36, 47]. Adding another small amino acid residue, as observed in PINK1 (Phe104Ala), promotes cleavage [47]. Conversely, mutations that have the ability to stabilize the helix confer cleavage-resistance, for example, Arg98Phe [40], Gly107Leu or Gly109Leu [41], and Pro95Ala [47]. Interestingly, PINK1 (Pro95Ala) still binds to PARL [47], suggesting that recognition of substrate and cleavage by PARL are separable. In this regard, it is noteworthy that Pcp1, a yeast orthologue of PARL, may recognize sequence regions outside of the TM domain when it cleaves Mgm1, one of the few known substrates of Pcp1 in yeast [48].

### Retro-translocation and N-end rule-dependent degradation of cleaved PINK1

Several groups have noticed that the 52 kDa form of PINK1 is unstable compared to full-length PINK1, and stabilized by proteasome inhibitors such as MG132 and Epoxomycin [9, 32, 35, 37]. Proteasomal elimination of the 52 kDa PARL-cleaved PINK1 is consistent with studies localizing the 52 kDa form of PINK1 in the cytosol [32, 37], indicating that cleaved PINK1 is retro-translocated into the cytosol before degradation by the proteasome.

Subsequently, Yamano et al. analyzed the detailed mechanisms of the degradation of cleaved PINK1 [49]. As mentioned above, PINK1 is cleaved by PARL after Ala103, yielding Phe104 as the new N-terminal amino acid. It is known that certain N-terminal amino acids function as signals for ubiquitination, so-called N-degrons, which are divided into type-1 (basic) and type-2 (bulky hydrophobic) [50]. Thus, Phe is categorized as one of the type-2 N-degrons. Consistent with this, when Phe104 is mutated to Met, a stabilizing residue, both PARL-mediated cleavage and subsequent retro-translocation to the cytosol occurs normally, but the 52 kDa cleaved form of PINK1 (Phe104Met) becomes resistant to degradation. The E3 enzymes UBR1, UBR2, and UBR4, which recognize type-2 degrons [51], are responsible for degradation of cleaved PINK1. Thus, in healthy mitochondria, PINK1 is cleaved by PARL soon after import and retro-translocated to the cytosol, where it is subjected to constitutive degradation via the N-end rule proteasome pathway (Fig. 3).

The model seems to be simple at a glance, but several questions still exist. It is expected that extended polypeptides with lengths from 50 amino acids to 60 amino acids can span both the OMM and IMM [52, 53]. Indeed, some in vitro experiments can be interpreted to indicate that PARL may cleave PINK1 on the IMM while the C-terminal domain of PINK1 still remains in the cytosol [49]. We speculate that PARL might obtain access to an import-intermediate of PINK1 during membrane translocation. However, it remains elusive how PARL can cleave proteins still within the import channel complex. Perhaps PINK1 laterally exits Tim23 to reach PARL while still retained in the OMM through the TOM channel.

### Possible regulators of PINK1 cleavage and lessons from yeast

PARL-mediated cleavage of PINK1 seems to require energetically driven import through the Tim23 complex. In addition, other possible regulators of PINK1 cleavage have been reported. For example, a *Drosophila* study showed the possible involvement of LON protease, one of the matrix-localized proteases, in PINK1 cleavage and/or degradation [54]. More recently, a membrane scaffold protein, SLP2, and a subunit of IMM-localized i-AAA protease, YME1L, were reported to facilitate PARL-mediated PINK1 processing [55]. BN-PAGE analysis showed that PARL consists of a large complex (approximately 2 MDa) with SLP2 and YME1L, suggesting that PARL protease activity itself and/or its substrate recognition are regulated by this complex. In another report, it was shown that knockdown of AFG3L2 attenuates the cleavage of PINK1, resulting in the accumulation of the MTS cleaved form [42], as observed in PARL KO MEFs [40]. AFG3L2 is a subunit of another IMM-resident protease, the so-called m-AAA protease [56], although it remains elusive whether m-AAA protease can directly cleave PINK1 or assists the PARL-mediated cleavage. The m-AAA protease can also form hetero-oligomers, which contain SPG7 in addition to AFG3L2, but the attenuation of PINK1 cleavage was not observed in SPG7 knockdown [42].

It is interesting that both i-AAA and m-AAA were reported as possible regulators of PINK1 cleavage because i-AAA and m-AAA belong to the same ATP-dependent AAA protease family, which have their active sites oriented towards the IMS or matrix, respectively [56]. However, at the same time, it is known that i-AAA and m-AAA are responsible for the degradation of damaged IMM-localized proteins, such as oxidized OXPHOS components [56]. In this regard, we recently identified that PINK1 import (and its cleavage) could be attenuated in response to the accumulation of unfolded/aggregated mitochondrial proteins [57, 58] (see Section 7). The loss of mitochondrial proteases may, therefore, prevent PINK1 proteolysis indirectly by leading to misfolded protein accumulation. Therefore, careful interpretation of how experimental removal of proteases yields PINK1 accumulation is necessary.

**Fig. 4.**
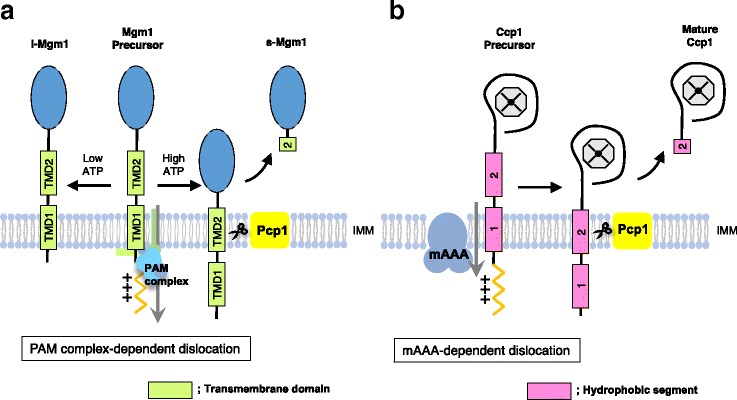
Pcp1-mediated cleavage of mitochondrial proteins in yeast. Pcp1, a PARL orthologue in yeast, is known to cleave Mgm1 **a** and Pcp1 **b**. The cleavage site of Pcp1 resides in the second hydrophobic segment of these substrates. For Pcp1-mediated cleavage of Mgm1 and Ccp1, some proteins (PAM complex and m-AAA, respectively) are involved in the membrane dislocation of substrates for presenting cleavage sites to Pcp1. This membrane dislocation process consumes ATP

Understanding the yeast PARL orthologue, Pcp1, may inform PARL-mediated PINK1 cleavage. For Pcp1, two substrates have been identified, Cytochrome C peroxidase (Ccp1), a heme-binding ROS scavenger [59], and Mgm1, the dynamin-related GTPase involved in mitochondrial fusion [43, 60, 61] (Fig. 4). Mgm1 has a 36 amino acid long N-terminal MTS followed by two hydrophobic segments, TMD1 and TMD2. Pcp1 cleaves the sequence within TMD2 (amino acids 156–169), but Pcp1-mediated cleavage of TMD2 is highly affected by the hydrophobicity of TMD1 (amino acids 94–111) [62]. When the activity of the PAM import motor was suppressed by ATP depletion or mtHSP70 deletion, Pcp1-mediated cleavage was severely retarded. From these results, Herlan et al. concluded that after the N-terminal region of Mgm1 passes through the Tim23 complex, a functional import motor is necessary to drive further translocation until the TMD2 reaches the IMM, allowing Pcp1 access to TMD2 (Fig. 4). Pcp1-mediated cleavage of Mgm1 requires the PAM complex motor activity, whereas Pcp1-mediated cleavage of Ccp1 requires m-AAA [63]. Ccp1 also has two hydrophobic segments after an 18 amino acid long N-terminal MTS. As observed in the case of Mgm1, the hydrophobicity of the first segment of Ccp1 affects Pcp1-mediated Ccp1 cleavage that occurs in the second hydrophobic segment of this protein. AAA proteases, including m-AAA and i-AAA, are known to have unfoldase activity in addition to protease activity [56]. Intriguingly, ATP-consuming unfoldase, but not protease, activity of m-AAA is required for Pcp1-mediated Ccp1 cleavage. From these observations, Tatsuta et al. [63] concluded that m-AAA mediates some degree of membrane dislocation of Ccp1 by using its unfoldase activity, and correctly positions Ccp1 within the IMM to allow cleavage (Fig. 3). These yeast studies emphasize the importance of the cooperative function of a membrane dislocator for PARL-mediated intramembrane proteolysis, but also yield the open question of whether similar mechanisms function in PINK1 cleavage.

### Other substrates of PARL in mammals

In addition to PINK1, other substrates of PARL have been reported in mammals, including the IMM-resident proteins PGAM5 and Smac/DIABLO [64, 65]. Unlike PINK1, these proteins are not readily exported into the cytosol after PARL-mediated cleavage, but specifically translocate into the cytosol during apoptosis. Both of these bind to anti-apoptotic IAP family proteins through their N-terminal motif, the so-called IAP binding motif, that is exposed by PARL-mediated cleavage, and function as apoptosis promoters [65, 66].

PGAM5 is an IMM-resident Ser/Thr protein phosphatase that functions in several stress responses, including apoptosis [67]. In contrast to PINK1, which is cleaved in healthy mitochondria, PGAM5 is cleaved by PARL in damaged mitochondria only after they lose ΔΨm [64]. Immunoprecipitation analysis revealed that PGAM5 binds to PARL in a time-dependent manner during treatment with the mitochondrial uncoupler CCCP, whereas PINK1 reciprocally dissociates from PARL, indicating that PARL cleaves different substrates, a kinase and a phosphatase, depending on the health status of mitochondria. However, the physiological significance of this phenomenon has not been elucidated. Intriguingly, SLP2 and YME1L, which seem to have supporting roles in PARL-mediated PINK1 cleavage, were reported to have inhibitory effects on PARL-mediated PGAM5 cleavage. Like SLP2, another mitochondrial membrane organizer protein, Prohibitin, was reported to inhibit the cleavage of OPA1, the mammalian homolog of Mgm1 [68], indicating the possible importance of the mitochondrial lipid compartment for mitochondrial protease-mediated proteolysis [69]. Future work is expected to better clarify the mechanisms by which SLP2 and YME1L can regulate PARL-mediated differential cleavage of PINK1 and PGAM5.

## Stress-dependent PINK1 accumulation on the OMM

### Full length PINK1 accumulated on the OMM recruits Parkin

Early studies showed that cleaved PINK1 accumulates upon proteasome inhibition and that PINK1 cleavage is inhibited upon treatment with mitochondrial uncouplers [34, 37]. In the former case, cleaved PINK1 is mainly observed in the cytosol, but in the latter case, full-length PINK1 is observed on mitochondria. However, the precise location of the full-length form of PINK1 within mitochondria and, importantly, its role there was unknown at that time. Shortly thereafter, Narendra et al. reported that cytosolic Parkin is recruited to damaged mitochondria upon treatment of cells with mitochondrial uncouplers, and promotes autophagic degradation of damaged mitochondria [7], linking sets of data related to PINK1 and Parkin to one another. Biochemical assays using isolated mitochondria revealed that the full-length form of PINK1 accumulates on the OMM in response to ΔΨm loss [8, 9, 40]. And this OMM-localized full-length form of PINK1 was shown to recruit cytosolic Parkin to damaged mitochondria [8–11, 70]. These studies provided a molecular mechanism for the genetic interaction between PINK1 and Parkin that was revealed in *Drosophila* [3, 4].

### Import arrest induced by inactivation of the N-terminal MTS of PINK1

Recent studies reveal that PINK1 accumulation in the OMM can be induced in several ways in addition to ΔΨm loss. For example, knockdown of MPPβ, the catalytic subunit of the dimeric matrix protease MPP, is reported to induce the PINK1 accumulation in the OMM [42]. In addition, Okatsu et al. reported that PINK1Δ34 (an N-terminal MTS-deleted form of PINK1) is not imported into the IMM, but mainly localized at the OMM [33]. A similar observation was observed with N-terminally tagged PINK1. Generally, N-terminal tagging inhibits the translocation of precursors carrying an N-terminal MTS to mitochondria. However, N-terminally Myc tagged PINK1 (Myc-PINK1) still localizes to mitochondria [32], but in this case it targets to the OMM [33]. PINK1 accumulated in the OMM acquires kinase activity through auto-phosphorylation (see Section 6). Consistently, PINK1Δ34 and Myc-PINK1 were phosphorylated under steady state conditions, indicating that these PINK1 forms become active. N-terminally Flag-tagged PINK1 has similar properties. The Flag tag contains several negatively charged Asp residues. Indeed, N-terminal fusion of five Asp residues to PINK1 ([Asp]-PINK1) also yields a constitutively active form that resides in the OMM. These results suggest that inactivation of the N-terminal MTS of PINK1 alone is sufficient to promote PINK1 localization in the OMM [33].

### The domain required for PINK1 retention in the OMM

When translocation within the Tim23 complex halts due to ΔΨm loss, most precursor proteins are exported into the cytosol, whereas PINK1 is retained in the OMM. In several reports, alkaline extraction of isolated mitochondria indicates that PINK1 accumulated in the OMM associates with the membrane as tightly as OMM-localized membrane spanning proteins [35, 42]. A PINK1 mutant deleted in the TM domain (amino acids 94–110; PINK1ΔTMD) can accumulate in the OMM in response to ΔΨm loss [33], suggesting that PINK1 OMM localization depends on another domain. Okatsu et al. found that PINK1 has a weak hydrophobic segment just N-terminal to the TM domain (amino acids 70–95) based on detailed structural prediction analysis [33]. When MTS function was inhibited (CCCP pre-treatment or the insertion of negatively charged Asp residues at the N-terminus), PINK1 (amino acids 1–90) was sufficient for PINK1 OMM localization. From these results, Okatsu et al. speculated that the newly identified hydrophobic segment around 70–95 amino acids may act as an OMM retention signal, and named this domain “outer mitochondrial membrane localization signal” (OMS) [33] (Fig. 2).

As mentioned before, inactivation of the MTS can promote OMM localization of PINK1, suggesting that competition between MTS and OMS might determine PINK1 localization within mitochondria. As the Tim23 complex pulls the MTS into the matrix, what counteracts this and recognizes the OMS to promote retention of PINK1 in the OMM? Also, why is the OMS domain of PINK1 not active under steady state conditions? Several such issues remain unresolved. The observations that knockdown of Tom40 inhibits the OMM localization and activation of all PINK1 mutants lacking MTS activity [33] suggest that at least Tom40 is required for the OMM retention of PINK1.

## PINK1 high molecular weight complex on the OMM

**Fig. 5.**
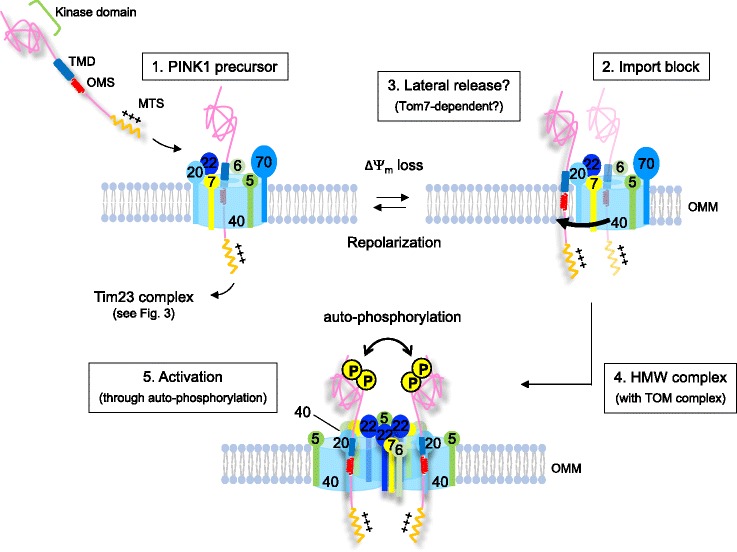
High molecular weight (HMW) complex formation of PINK1. PINK1 forms a high molecular weight (HMW) complex with the TOM complex on the OMM in response to ΔΨm loss. Tom7, an accessory subunit of the TOM complex, may be involved in the lateral release of PINK1 into the OMM. The TOM complex is considered to provide a location for the activation of PINK1 kinase activity by facilitating the correct orientation of dimeric PINK1 to allow auto-phosphorylation *in trans*. At the same time, HMW complex formation with the TOM complex may allow rapid PINK1 re-import when mitochondria are repolarized to halt mitophagy

BN-PAGE analysis revealed that OMM-accumulated full-length PINK1, but not the 52 kDa cleaved PINK1, forms a high weight molecular (HWM) complex with the TOM complex [71, 72] (Fig. 5). The TOM complex itself appears around 500 kDa, whereas the PINK1 HMW complex appears around 720 kDa. At least three subunits of TOM complex, Tom20, Tom22, and Tom40, have been commonly identified as the components of the PINK1 HMW complex. An immunoprecipitation assay we employed using cross-linkers revealed that OMM-accumulated PINK1 crosslinked with Tom20, but not Tom40 [71]. Therefore, it can be speculated that PINK1’s association with the TOM complex does not result from stalled import upon ΔΨm loss, but might be laterally released from the Tom40 channel to the OMM, still associated with Tom20 [71]. This hypothesis is consistent with the previous observation that accumulated full-length PINK1 is tightly associated with the OMM, presumably through the OMS domain.

Then how is full-length PINK1 laterally released from the TOM complex? Lateral release from the TOM complex itself is not well understood, and it is only shown to occur with model proteins in yeast [73]. A possible lateral release mechanism of PINK1 may involve Tom7. Tom7 is a small accessory molecule of the TOM complex, and the most recent cryo-EM analysis of the TOM complex reveals that Tom7 seems to associate tightly with the Tom40 channel [74]. We identified Tom7 as an essential factor for ΔΨm loss-induced PINK1 accumulation in a full genome RNAi screen of Parkin translocation [75]. In vitro import assays using isolated mitochondria derived from Tom7 KO HeLa cells revealed that Tom7 is not required for normal PINK1 import under steady state conditions, whereas in Tom7 KO HeLa cells, ΔΨm loss-induced PINK1 accumulation in the OMM was completely abolished, indicating that Tom7 somehow promotes PINK1 retention in the OMM (Fig. 5).

Why does PINK1 form a HMW complex with the TOM complex? So far, two possible reasons have been suggested. We previously showed that PINK1 re-import starts within minutes after wash-out of a mitochondrial uncoupler [9, 71], which results in the disappearance of PINK1 HMW complex and Parkin dissociation from the mitochondria [71]. Therefore, the PINK1 HMW complex formation with TOM complex may allow the rapid re-import of PINK1 to rescue repolarized mitochondria from mitophagy. Another model is that the TOM complex may denote the location of sites of PINK1 kinase activity stabilization on the mitochondrial surface [72], which may signal to the cytosol indicating the subdomains of the matrix where damage exists [57, 58]. In the following section, we will summarize the reported mechanisms of PINK1 kinase activation.

## The activation of PINK1 kinase activity on the OMM

### PINK1 is activated through auto-phosphorylation

PINK1 has a Ser/Thr kinase domain, from amino acids 156–509, with a high degree of homology to the Ser/Thr kinase of the Ca^2+^/calmodulin family [1] (Fig. 2b). Most of the PD-associated mutations reside within the protein kinase domain of PINK1 (Fig. 2b), emphasizing the importance of PINK1 kinase activity for its protective role against PD. PINK1 kinase activity has been detected by using recombinant proteins, and the PINK1 kinase dead (KD) mutant, PINK1 (K219A/D362A/D384A), was created in one of these studies [76]. These amino acid residues are predicted to be important for orienting ATP and carrying out the phospho-transfer reaction to acceptor residues on the substrates.

The phosphorylation status of PINK1 from cell lysates is able to be monitored using phos-tag gels [77]. ΔΨm loss-induced accumulated full-length PINK1 shows a clear mobility shift by phos-tag western blotting. By using this method, Okatsu et al. identified two PINK1 auto-phosphorylation sites, Ser228 and Ser402, that are required for the activation of PINK1 kinase activity [77] (Fig. 2b). A PINK1 KD mutant expressed in PINK1 KO MEFs lacked phosphorylated bands on phos-tag western blotting, indicating that it is auto-phosphorylated. When PINK1 wild type and PINK1 (G409V), one of the pathogenesis-associated mutants that lack kinase activity, were co-expressed, PINK1 (G409V) also was phosphorylated, suggesting that PINK1 can phosphorylate itself *in trans*. When the identified auto-phosphorylation sites were mutated to Ala, PINK1 (S228A/S402A) did not display phosphorylated bands on phos-tag western blotting. Several kinases are known to share a common mechanism of activation by trans-phosphorylation of their activation loops, and PINK1 seems to utilize the same mechanisms, as Ser402 resides in the activation loop (amino acids 384–417; Fig. 2b). It was previously shown that PINK1 recruits Parkin in a kinase activity-dependent manner (for example, PINK1 KD mutant cannot rescue Parkin recruitment in PINK1 KO MEFs) [8, 9]. Consistent with this, the PINK1 (S228A/S402A) mutant cannot rescue Parkin recruitment in PINK1 KO MEFs, but a PINK1 (S228D/S402D) phosphorylation mimetic PINK1 mutant can. These results clearly suggest that PINK1 auto-phosphorylation upon ΔΨm loss is essential for Parkin recruitment to damaged mitochondria [77].

### PINK1 HMW complex and auto-phosphorylation

As mentioned above, PINK1 forms a HMW complex with the TOM complex on the OMM upon mitochondrial depolarization. The TOM complex has been considered to contain two or three Tom40 channels [30, 74, 78]. Okatsu et al. showed that the PINK1 HMW complex contains two PINK1 molecules [72]. In addition, two-dimensional electrophoresis (first, BN-PAGE; second, phos-tag SDS-PAGE) revealed that PINK1 in the HMW complex is in the phosphorylated form. Considering this together with the fact that PINK1 auto-phosphorylates *in trans* [77], Okatsu et al. suggest a model in which the TOM complex might assist in orienting the dimeric PINK1 to facilitate intermolecular phosphorylation [72] (Fig. 5), which is another possible reason why PINK1 forms HMW complex with TOM complex. Lazarou et al. showed that the PINK1 that forms a complex with TOM of around 720 kDa only occupies a small fraction of the total TOM complexes (approximately 5% of the total abundant TOM complexes at around 520 kDa) [71]. When PINK1 only occupies 5% of the TOM complexes, it is curious how the second PINK1 docks to the relatively sparse previously PINK1-occupied TOM complexes rather than docking to unoccupied TOM complexes. Regional concentration of productive TOM/PINK1 interactions as discussed below may foster TOM subset assembly of PINK1 dimers and may also relate to TOM/TIM23 super-complex formation, perhaps also involving PARL.

## Mitochondrial proteotoxic stress-induced PINK1 activation

Our recent study revealed that PINK1 import arrest and subsequent activation also occurs in response to mitochondrial proteotoxic stress, which is induced by the forced expression of misfolded aggregates of mitochondrial-localized mutant ornithine transcarbamylase (ΔOTC) [57]. ΔOTC-induced PINK1 activation recruits Parkin to mitochondria, which promotes the clearance of aggregated ΔOTC from mitochondria [58]. Intriguingly, ΔOTC-induced PINK1 accumulation is not accompanied by mitochondrial depolarization, indicating that PINK1 import arrest is also induced by an unidentified mechanism [57]. In addition, unlike mitochondrial uncouplers, ΔOTC-induced PINK1 accumulation and Parkin recruitment occur on focal spots on mitochondria that are proximal to ΔOTC aggregates within the mitochondrial network [58]. This focal activation of PINK1/Parkin allows the selective clearance of ΔOTC aggregates, but not wild-type OTC, from mitochondria with the help of the mitochondrial fission factor Drp1. Insights into the detailed mechanisms of PINK1 import regulation under proteotoxic stress are expected in the future.

## In vivo relevance and potential for drug discovery

Although dopaminergic neuronal loss is not observed in either PINK1 or Parkin KO mice, we recently found that when Parkin is deleted in mice that express a proof-reading-defective version of the mtDNA polymerase (POLG), the so-called Mutator mouse, certain features of PD pathogenesis develop, including dopaminergic neuron degeneration and motor defects [79]. Of note, quantitative mass spectrometry showed that phospho-Ser65 ubiquitin levels increased in the brains of Mutator mice relative to wild-type mice. In addition, by using a recently developed antibody against phospho-Ser65 ubiquitin, phosphorylated ubiquitin was found to be increased in brain samples of patients harboring pathogenic PD mutations [80]. Because PINK1 is the only known kinase that can phosphorylate Ser65 on ubiquitin, these observations support the notion that PINK1/Parkin pathway activation occurs in vivo under pathophysiological conditions.

The most deleterious clinically relevant mutations in PINK1 occur in its kinase domain, and these mutations reduce the PINK1 kinase activity. Thus, the pharmacological activation of PINK1 mutants with reduced kinase activity may be one therapeutic approach for certain forms of PD. For example, one study describes a novel means of activating PINK1 kinase activity by using the ATP analog N^6^-furfuryl ATP (kinetin triphosphate, KTP) [81]. PINK1 accepts the KTP with higher catalytic efficiency than its endogenous substrate, ATP, and treating cells with the metabolic precursor of KTP, kinetin, enhances Parkin recruitment to depolarized mitochondria in a PINK1-dependent manner. Further validation of kinetin activity, especially in vivo, is expected. Although the activation of PINK1 kinase activity is closely related to its import regulation, as discussed above, there are no reports of small molecules targeting PINK1 import so far, although attempts are being made in this promising new direction [82].

## Future directions for stress-dependent import regulation of PINK1

In this review, we focus on the molecular mechanisms of mitochondrial stress-dependent PINK1 activation. Mitochondrial depolarization is signaled to the cytosol through PINK1 import arrest. PINK1 activation is also somehow induced by mitochondrial proteotoxic stress. Intriguingly, a recent *Caenorhabditis elegans* study identified another protein, ATFS-1, whose mitochondrial import is blocked in response to the mitochondrial proteotoxic stress [83]. ATFS-1 is then translocated into the nucleus where it promotes the expression of mitochondrial chaperones and proteases to remove the damaged proteins in mitochondria. These results suggest that stress-dependent mitochondrial import regulation is an evolutionarily conserved strategy to convey a sign of mitochondrial damage to the cytosol.

In addition to PINK1 phosphorylation of ubiquitin on mitochondria, recent studies also reveal potential PINK1 substrates in other compartments (for example, an OXPHOS component in the IMM [84] and the PGC1α regulator PARIS in the cytosol [85]). Thus, further studies on how the PINK1 kinase is activated in compartments other than the OMM are warranted. The discovery of stress-dependent PINK1 import regulation yields a novel sensing mechanism of mitochondria. It is expected that future studies will identify other examples of this elegant system and explore in greater detail drugs to promote mitophagy by targeting PINK1 or PINK1 import.
